# Practical issues in the management of sleep, anxiety, and mood disorders in primary headaches

**DOI:** 10.1055/s-0045-1809881

**Published:** 2025-07-02

**Authors:** Patrick Emanuell Mesquita Sousa-Santos, Mario Fernando Prieto Peres

**Affiliations:** 1 Universidade Estadual Paulista, Botucatu SP, Brazil.; 2 Universidade de São Paulo, Faculdade de Medicina, Hospital das Clínicas, Instituto de Psiquiatria, São Paulo SP, Brazil.; 3 Hospital Israelita Albert Einstein, São Paulo SP, Brazil.

**Keywords:** Headache Disorders, Migraine Disorders, Sleep Wake Disorders, Anxiety Disorders, Mood Disorders

## Abstract

There are many conditions associated with primary headaches, including mood (depression), anxiety, and sleep disorders, which are highly prevalent in the general population and in tertiary headache centers. We call this set of symptoms migraine, anxiety, mood, and sleep (MAMS). The presence of one or more of these symptoms can alter the clinical course and represent a practical challenge. In the present study, we aimed to describe a practical approach to treat these symptoms frequently associated with headaches. Diagnostic aspects and secondary causes were addressed as well as the shared mechanisms and cause and effect relationship. Finally, we commented on the therapeutic approach used to treat these symptoms.

## INTRODUCTION

Headache disorders rank prominently in global morbidity, positioned as the fifth leading cause of disability-adjusted life-years (DALYs) among adults, a testament to their significant public health impact, as the Global Burden of Disease study underscores. 1 In young women aged 10 to 24 years, headache disorders ascend to the primary cause of DALYs, alarmingly accompanied by depressive and anxiety disorders in the top 3. 1 Over 90%, of these debilitating headache disorders are primary headaches (PHs), which include common forms such as tension-type headache (TTH), migraine, and cluster headache (CH). Among these, migraine significantly overshadows others regarding its disability contribution, with a striking prevalence of 15.2% in certain populations, as observed in demographic studies within Brazil. 2 Despite their considerable social repercussions and economic costs, PHs have historically been neglected in global health policies, a trend that belies their societal importance.

Complicating the clinical landscape of PHs is the frequent co-occurrence of additional conditions, adding layers to their medical complexity. A broad meta-analysis, encapsulating research from 2000 to 2020, emphasized the prevalence of comorbidities in primary headache sufferers, particularly mood disorders (including depression), anxiety disorders, and sleep disturbances. 3 Migraines have been a focal point in comorbidity research within PHs, given their significant psychiatric overlap, a phenomenon recognized since the exploratory studies of the 1970s. 4

Approximately half of individuals with chronic migraine also suffer from concurrent psychiatric conditions, with higher prevalence in specific demographic and clinical subgroups, such as women, patients with chronic migraine, and those experiencing migraine with aura. 5 6 7 The Cameo study delineates the intricacies of chronic migraine profiles, revealing a direct correlation between heightened disability and the number of comorbid conditions. 8

Additionally, sleep disorders consistently manifest in conjunction with TTH and migraine, often exacerbating the individual's overall condition. 9 10 The clinical observations underline a deterioration in sleep quality both prior to and during headache episodes, with numerous therapies inadvertently influencing sleep patterns. 11 The predicament is particularly acute in CHs, which are associated with distinctive sleep disturbances partly due to their nocturnal onset. 12 Furthermore, CHs increased almost three times the odds of depression. 12

Thus, we chose mood disorders, anxiety, and sleep disorders as the pillars of associated conditions due to their high prevalence both in the general populace and within specialized headache centers. We call this set of symptoms migraine, anxiety, mood, and sleep (MAMS). The existence of one or multiple of these disorders not only complicates the clinical trajectory but also presents substantial practical management challenges. The themes addressed in this manuscript were carefully selected based on the extensive experience of the senior author, MFPP. The treatment landscape is intricate, with many medications affecting these conditions in diverse ways, potentially indicating shared therapeutic targets and pathophysiological mechanisms. 13 We aimed to describe in the present study a practical approach to the triad of conditions most associated with primary headaches—constituting the MAMS spectrum—based on extensive clinical observation and experience: anxiety, mood, and sleep disorders.

## DIAGNOSTIC ASPECTS

Diagnosing MAMS is often complicated due to symptom overlap. Patients may present with comorbid conditions, making it difficult to identify the primary disorder or understand the causal pathways. Misdiagnosis or incomplete diagnosis can lead to ineffective treatment plans, emphasizing the need for comprehensive assessment tools and interdisciplinary communication to accurately map a patient's health profile.

Stigma continues to be a barrier in the management of MAMS, particularly concerning mental health components. Patients may be reluctant to seek help or disclose symptoms, fearing societal judgment or misunderstanding. This impediment can delay diagnosis, diminish the efficacy of interventions, and exacerbate isolation or distress. Educating patients, families, and the public is essential to dismantle misconceptions and encourage a supportive, empathetic approach to these health concerns.

## SCREENING AND EVALUATION STRATEGIES

The convergence of sleep, mood, and anxiety disorders with PHs requires nuanced evaluation and management strategies. Two primary approaches emerge:

- referral to specialists for each condition, or
- comprehensive management by a single clinician.

While specialist referral can offer in-depth management, potential drawbacks include treatment delays, diminished patient adherence, and escalated costs. Conversely, management by a single clinician enhances treatment immediacy, adherence, and fortifies the patient-doctor relationship, albeit demanding continual clinician education and heightened vigilance in complex cases or non-responsive scenarios.

### Symptom-based approach versus full-blown diagnosis

The identification of the major associated conditions is crucial for practical reasons, including treatment selection. The diagnostic standards rely on manuals like the Diagnostic and Statistical Manual of Mental Disorders (DSM), International Classification of Sleep Disorders (ICSD), International Classification of Diseases (ICD), and the International Classification of Headache Disorders (ICHD). However, research indicates that clinicians may not always adhere to these criteria when diagnosing and deciding on treatments. 14 Numerous studies employ validated questionnaires to determine the presence of depression, anxiety, and sleep disorders, as well as to assess treatment responses. Prominent among these questionnaires are the 7-Item General Anxiety Disorder (GAD-7) assessment, Epworth Sleep Scale (ESS), Beck's Depression Inventory (BDI), Insomnia Severity Index (ISI), and the Mood Disorders Questionnaire (MDQ). 14 15 Given the diversity of criteria, an alternative is the symptom-based approach, which is gaining traction in mental health research. This approach seeks to address the limitations of traditional classification systems, which often group patients with varying symptom presentations and intensities under a single diagnostic label. 14

### Secondary causes

It is essential to consider secondary causes as potential underlying mechanisms for associated conditions. Factors such as head trauma, cerebrovascular events, systemic diseases, hormonal fluctuations, and environmental aspects may play a role in exacerbating headaches, as well as contributing to anxiety, mood disturbances, and poor sleep quality. Table 1 enumerates the secondary causes.

**Table 1:** Secondary causes of headaches/migraine-type, potentially associated with anxiety, mood disorders, and sleep disorders are shown

Cause	Etiology	DD	Complementary exams	Comments
Vascular	Subarachnoid hemorrhage/Aneurysms	M	Brain CT	Sudden onset headache, thunderclap headache
	Arterial dissections	M	Angio CT/ MRI arteriography	Sudden onset headache, cervical pain, and focal deficits
	Cerebral thrombosis	M	Angio CT/MRI	Seizures, papilledema, and encephalopathy/coma
	Stroke	MAMS	Brain CT/MRI	Neurological deficits
Infection	Meningitis	M MS	CSF examination CT/MRI	Fever and systemics symptoms
	Sinusitis		CT	
	HIV	MAMS	Serological tests	Opportunistic infections, weight loss
	Syphilis	MAMS	Serological tests CSF examination	
Traumatic	Concussion	M	Brain CT	Traum history
	Subdural hemorrhage	M	Angio CT/MRI	
Metabolic/Nutritional	Hyponatremia	MAMS	Electrolytes tests	Progressive symptoms
	Vitamin D, and B12	MAMS	Vitamin panel	History of abdominal surgery
	Iron and folate deficiency	MAMS	CBC, iron and folate dosage	Asthenia/fatigue
Neoplasm	Metastasis/cerebral tumor	MMS	Contrast Brain CT/MRI	Weight loss, age, personal or familiar history
Inflammation	Giant cells arteritis	M	Erythrocyte sedimentation rate	Onset > 50 years, jaw pain, vision problems
Endocrinal	Diabetes	MAMS	Glucose/HbA1C	We recommend the endocrine basic tests in all cases of MAMS
	Thyroid disorders	MAMS	TSH, FT4	
	Adrenal disorders	MAMS	Cortisol/ACTH	
Others	CSF leak	M	CSF examination	Orthostatic headache
	Pseudotumor cerebri	M	CSF examination	Papilledema, visual loss, diplopia, obesity
	Glaucoma	M	Ophthalmologic evaluation	Eye pain/red eye
	Substance abuse (as alcohol and drugs)	MAMS		Clinical history
	Multiple sclerosis	MAMS	MRI and CSF	Neurological deficits, optic neuritis

Abbreviations: ACTH, adrenocorticotropic hormone; Angio, angiography; CBC, complete blood count; CT, computed tomography; CSF, cerebrospinal fluid; DD, differential diagnosis; FT4, free thyroxin; HbA1C, glycosylated hemoglobin; MAMS, migraine, anxiety, mood disorders, and sleep disturbances; MRI, magnetic resonance imaging; TSH, thyroid-stimulating hormone.

Note: Isolated “M” indicates headache/migraine-type. The mnemonic “VITAMINS” can be helpful in considering various etiologies.

### Complementary exams

The current overreliance on imaging examinations poses potential issues, as it exposes patients to the inherent risks associated with these methods (such as radiation, contrast use, and incidental findings) and can unnecessarily escalate healthcare costs. The criteria for recommending neuroimaging in headache outpatient centers remain a subject of debate, leading to the development of guidelines like the SNNOOP10 list for identifying red flags in headache cases. 15 However, the effectiveness of these guidelines is questioned due to the lack of prospective epidemiological studies on red flags and the relatively low incidence of many secondary headaches. 15

The identification of specific features in cases of excessive daytime sleepiness, such as habitual loud snoring, witnessed episodes of apnea/gasping/choking, hypertension, and obesity, may warrant the use of polysomnography due to the risk of obstructive sleep apnea. 16 In practical settings, insomnia associated with headache, anxiety, and mood disorders that are refractory to clinical treatment (both non-pharmacological and pharmacological) should be further investigated with polysomnography.

Laboratory testing for reversible secondary causes, such as thyroid dysfunction, iron deficiency, and vitamin deficiencies, are cost-effective and minimally-invasive. These conditions often manifest through non-specific symptoms, such as fatigue, and may coexist with headaches, insomnia, and symptoms resembling anxiety and mood disorders.

## DIAGNOSTIC HINTS

### Anxiety

Anxiety is typically characterized by excessive worry, often accompanied by irritability, difficulties with concentration, and agitation. 17 In managing anxiety, a symptom-based approach is recommended, extending beyond the confines of DSM-based psychiatric diagnoses such as generalized anxiety disorder (GAD), obsessive-compulsive disorder (OCD), posttraumatic stress disorder (PTSD), panic disorders, and phobias. Anxiety should be delineated as either an excessive worry, an inability to control worries, or unrealistic fear, in contrast to irritability, which aligns more closely with mood-related symptoms.

Patients suffering from migraines concomitant with anxiety and depression are more prone to catastrophization, a psychological tendency that can significantly alter pain perception. 18 Anxiety is also linked to cephalalgiaphobia—the excessive fear or concern about developing a headache. 19

It is important to recognize that anxiety disorders may be secondary to various medications or clinical conditions, such as diabetes, arthritis, and cancer. Patients with anxiety often seek medical care for symptoms other than anxiety itself, with headaches being the most common complaint, followed by palpitations and chest pain. Additionally, anxiety frequently coexists with depression, substance overuse, and attention deficit hyperactivity disorder (ADHD). 6 17

### Mood

It is advisable to view mood on a spectrum ranging from negative aspects (such as depressive symptoms) to positive ones (including irritability, euphoria, and racing thoughts). This aligns with the concept of the bipolar spectrum. Diagnostically, patients may meet the criteria for a depressive episode or bipolar depression. Notably, ∼ 40% of individuals with migraines also experience depression, 20 and there is an increased likelihood of individuals with depression reporting headaches, indicating a bidirectional relationship. 20 Previous research has identified depression as a common comorbidity in migraine sufferers, with those experiencing CHs having a 3-fold increased likelihood of developing depression. 6

Mood disorders can arise secondary to specific medications or the use of recreational drugs (substance/medication-induced depressive disorder), as well as due to medical conditions like hormonal changes. Various factors contribute to mood disorders, with chemical imbalances in neurotransmitters being a primary suspect (refer to 'shared mechanisms' below). Stressful life events, such as death, divorce, or trauma, can also precipitate depression, particularly in individuals with a genetic predisposition.

There is evidence of potential overlapping neurobiological mechanisms between PHs and mood disorders, particularly bipolar disorder (BD). During the depressive phase of BD and between attacks in migraine patients, serotoninergic activity is observed to decrease. Dopamine receptor antagonists, such as chlorpromazine and metoclopramide, are employed in treating migraine attacks. Similarly, many medications primarily used for depression and BD are also considered first-line in the prevention of migraines. 21

### Sleep deprivation

**Figure 1:**
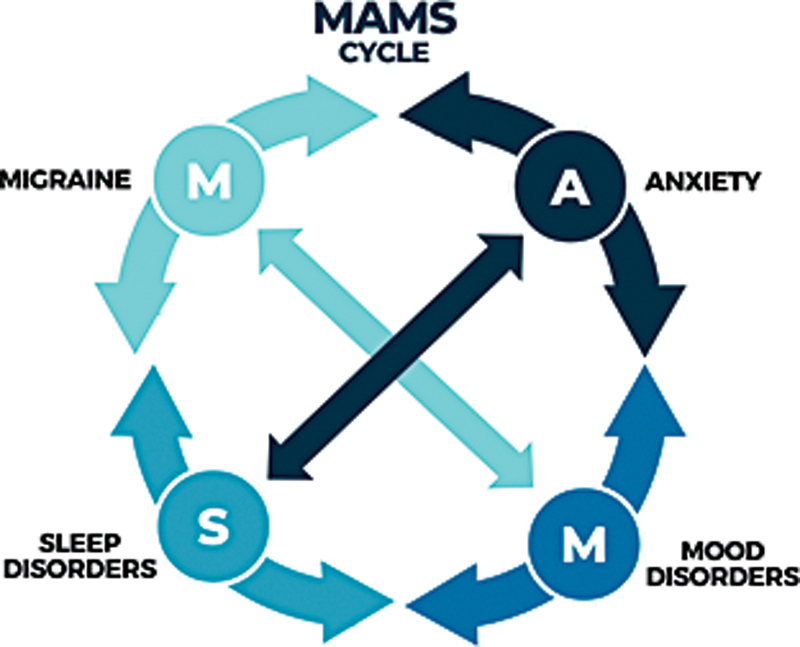
Abbreviations: BD, bipolar disorder; RLS, restless legs syndrome; REM, rapid eye movement. Algorithm for the diagnostic reasoning of sleep disorders.

A detailed history focusing on sleep patterns is essential. Patients who fall asleep quickly but wake up feeling tired or experience daytime sleepiness are likely suffering from sleep deprivation. It is well-established that sleep deprivation can trigger headache attacks in individuals with migraine. 22 Nonrestorative sleep may result from insomnia, sleep apnea, fragmented sleep, or other sleep disorders. Factors such as obesity, anatomical abnormalities, snoring, and cardiovascular risks necessitate a polysomnography test to evaluate for sleep apnea. Patients with obstructive sleep apnea often experience frequent headaches in the morning or upon awakening, which are related to hypoxemia and hypercapnia. 11 However, not every sleep complaint warrants a sleep study; in most cases, clinical history alone can identify the underlying issue. Figure 1 provides a guide for addressing sleep problems in patients with headaches.

Sleep disturbances, including both insufficient and excessive sleep, can be secondary to various factors such as allergies, upper respiratory infections, poor sleep hygiene, nocturia, chronic pain—including persistent headaches and continuous lower back pain—and stress or anxiety. Other notable sleep disorders include parasomnias (such as sleepwalking), narcolepsy characterized by daytime sleep attacks, and restless legs syndrome, which involves an overwhelming urge to move the legs, predominantly occurring at night.

## SHARED MECHANISMS IN PATHOPHYSIOLOGY

Numerous neurotransmitters, hormones, and neuropeptides play a crucial role in the pathophysiology of various conditions and serve as therapeutic targets common to mood disorders, anxiety, sleep disorders, and PHs. These shared biochemical elements underline the interconnected nature of these disorders and offer insights into potential integrated treatment approaches.

### The role of melatonin

Melatonin, a hormone synthesized in the pineal gland predominantly during the night, plays a crucial role in regulating the circadian rhythm by facilitating the onset and maintenance of sleep. 23 The hypothalamus modulates the release of melatonin in response to variations in light exposure (refer to the hypothalamus section for more details). Melatonin has been employed as a preventative treatment for migraine and other PHs, including hemicrania continua, cluster headache, and hypnic headache. 24 25 The exact mechanism by which melatonin alleviates headaches is not entirely clear, but it is believed that its primary action targets the hypothalamus. 23

### Serotonin and its implications

Serotonin (5-hydroxytryptamine, 5-HT) is a key neurotransmitter significantly involved in both mood disorders and migraines, as evidenced by the observed increase in serotonin metabolites in urine during migraine attacks. 26 In cases of depression, a reduction in 5-hydroxyindoleacetic acid, the primary metabolite of serotonin, is noted in the cerebrospinal fluid. This supports the monoaminergic model which posits a depletion of serotonin as one of the underlying causes of depression. 27 Serotonin also plays a role in sleep as it is involved in the production pathway of melatonin. 26 Therapeutically, many treatments for headaches (such as triptans and ditans) as well as for anxiety and mood disorders (including SSRIs and SNRIs) exert their effects through serotonergic mechanisms.

### Dopamine

Dopamine modulation plays a significant role in both the premonitory symptoms of migraines, such as yawning and dizziness, and in attack symptoms like nausea and vomiting. 11 Beyond migraines, dopamine is also implicated in the pathophysiology of CHs. 28 Its dysfunction is a key factor in parasomnias and sleep-related movement disorders, particularly restless legs syndrome (RLS). In mood disorders, dopamine system dysfunction is linked to symptoms of apathy and anhedonia, with studies highlighting increased striatal dopamine transporter binding in anxiety disorders. 11 29

### GABA and its inhibitory effects

The Gamma-aminobutyric acid (GABA) exerts an inhibitory effect on the nervous system and is crucial for the onset and maintenance of sleep. GABA release in the hypothalamus modulates pain pathways, and several medications, such as gabapentin (a GABA analogue) and benzodiazepines, act through GABAergic mechanisms. Neurofunctional studies have shown that thalamic trigeminovascular neurons containing GABA project to multiple cortical regions, potentially contributing to the modulation of migraine attacks. 28 30

### Glutamate's excitatory function

Glutamate, the primary excitatory neurotransmitter in the central nervous system, acts on N-methyl-D-aspartate (NMDA), AMPA, and kainate receptors. Numerous studies suggest there is glutamate involvement in the pathophysiology of PHs, particularly migraines, in relation to allodynia and central sensitization. 30 31 Topiramate, commonly used in migraine prevention, acts in part by inhibiting glutamate receptors. Similarly, ketamine, an NMDA receptor antagonist, has shown potential in reducing headache severity in chronic migraine and major depression patients, although evidence is still emerging. 31

## CGRP

The calcitonin gene-related peptide (CGRP), a potent vasodilator, is integral to the modulation of trigeminovascular responses and is central to migraine pathophysiology. It activates adenylyl cyclase and is present in over half of the neurons in the trigeminal ganglion. 32 Since the discovery of CGRP in 1982, numerous studies have linked this neuropeptide with PHs, such as migraines and CHs. 33 The development of monoclonal antibodies to block CGRP effects, approved by the Food and Drug Administration (FDA) in 2018 for migraine prevention, has been a significant advancement. 32 Currently, almost 1 million patients use these medications, with real-life studies reporting improvements in anxiety symptoms and both objective (assessed by polysomnography) and subjective sleep quality in patients using anti-CGRP therapies like erenumab, galcanezumab, and fremanezumab. 34 35

## THE CAUSE AND EFFECT RELATIONSHIP

**Figure 2:**
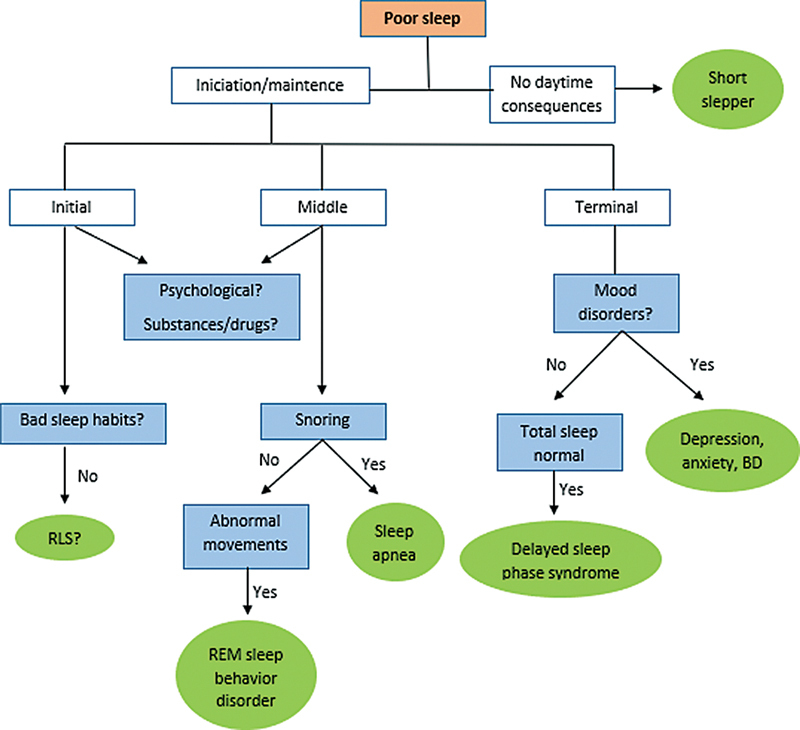
Interaction of migraine, anxiety, mood, and sleep (MAMS): Each factor interacts with the next in a vicious circle.

The major conditions associated with PHs—headaches, anxiety, mood, and sleep issues—are intricately interconnected, as illustrated in Figure 2 . These elements can mutually influence each other; for instance, headaches can exacerbate mood disorders, amplify anxiety, and disrupt sleep. Similarly, mood disturbances can intensify and prolong headaches, while anxiety can further worsen headaches, sleep quality, and mood. Sleep deprivation, in turn, can contribute to mood disorders, elevate anxiety levels, and trigger headaches.

It is crucial to interpret cross-sectional studies that show associations among these four disorders within the context of a cause and effect relationship. Only through longitudinal, prospective observation can we accurately determine if one disorder is the causative factor for another. While each of these four aspects has been individually studied in this manner, a comprehensive analysis of their collective impact has not yet been conducted. When a patient with PHs presents with additional sleep, anxiety, and mood issues, disentangling the sequence of onset can be challenging due to recall bias. However, it is essential to attempt this. A thorough assessment of the patient's family and personal history is also critical, as various genetic predispositions have been identified.

## STRATEGIES FOR TREATING MAMS

Even when one disorder is identified as the initial or causative factor in a patient's history, the optimal therapeutic approach involves treating all present symptoms and conditions.

The treatment plan begins with educating the patient about the complex interplay of the MAMS elements and addressing any fears related to potential secondary causes like tumors or aneurysms. Though the journey may be complex, it ultimately leads toward an improved quality of life. Patients should understand that while addressing all four conditions may present challenges, this comprehensive approach is likely to yield better results, improve prognosis, and explain previous treatment refractoriness due to the overlooked interconnection of these conditions.

In such scenarios, non-pharmacological strategies, including physical exercise and behavioral therapy, have all shown efficacy in improving each condition. Stress management, through mindfulness, yoga, or meditation, can significantly impact MAMS by alleviating symptom triggers. These practices promote relaxation, enhance coping mechanisms, and improve overall wellbeing. Instructing patients in sleep hygiene can mitigate sleep disturbances—key contributors to MAMS. The guidelines focus on creating a conducive sleep environment, establishing consistent sleep schedules, and managing dietary or behavioral factors that impinge on sleep quality.

The pharmacological management of MAMS is intricate due to the diverse neurochemical underpinnings and potential for drug interactions, especially when treating comorbid conditions. Selecting appropriate medication requires an understanding of the individual's complete health picture, including other prescriptions, to avoid adverse effects. Clinicians must be meticulous in monitoring patient responses and making necessary adjustments to the treatment plan.

Selecting medication for MAMS necessitates a holistic view of the patient's symptoms, co-occurring conditions, and potential interaction with existing therapies. Effectiveness, side effects, patient history, and preferences should inform this personalized approach, optimizing safety and adherence.

The decision regarding medication, whether to start with a single drug or a combination, poses a dilemma. The lack of randomized clinical trials examining all four conditions simultaneously means that evidence-based medicine offers limited guidance here. Table 2 depicts the potential effectiveness of various interventions across the four domains. Determining and discussing the matrix of symptoms/conditions versus treatment options with patients is critical.

**Table 2:** Comparison of the main interventions in different conditions associated with primary headaches

	Migraine pain headache	Anxiety	Mood	Sleep
*Pharmacological intervention*				
Muscle relaxant	++	−	−	++
Benzodiazepines	−/+	++	−	++
Melatonin	++	+	−	++
Beta blockers	++	+	–	−
Tricyclic antidepressants	++	++	+	++
SSRIs	+	++	++	−
SNRIs	++	++	++	−
Topiramate	++	−	–	+
Valproate	++	−	++	−
Pregabalin	−/+	++	−	++
Botulinum toxin	++	−	−	−
*Non-pharmacological intervention*				
Exercise	++	++	++	++
Meditation	++	++	++	++
Psychology	++	++	++	++

Abbreviations: SNRIs, serotonin-norepinephrine reuptake inhibitors; SSRIs, selective serotonin reuptake inhibitors.

Notes: ++, very useful; +, useful; −/+ , not very useful; −, not useful; –, risk of worsening.

Combinations of different drug classes may be necessary. Vigilance in managing side effects and potential drug interactions is crucial. Regular follow-ups, patient education about potential adverse effects, and coordination with other healthcare providers ensure a unified strategy in mitigating risks and enhancing patient comfort and compliance. Particular attention should be given to potential weight gain associated with many of these medications, especially in conditions like anxiety and mood disorders in which increased food intake is common. 36 37 Other side effects to consider include cardiovascular risks and decreased attention. Patients often harbor fears or resistance toward medication due to preconceived notions. Thus, fostering a strong patient-physician relationship is crucial for therapeutic adherence. Specific strategies, such as dietary and exercise plans, should be implemented to address concerns like weight gain. Furthermore, awareness of the nocebo effect, in which negative expectations can influence treatment outcomes, is vital. Care must be taken to balance informing patients about potential adverse effects without unduly emphasizing them. 38 39

### Spotlight on melatonin in treating MAMS

Melatonin has gained prominence as an effective treatment in the MAMS spectrum, particularly for its roles in sleep regulation and migraine prophylaxis. Beyond its known sleep-enhancing capabilities, melatonin offers significant benefits in managing associated MAMS disorders. The pivotal study by Gonçalves et al. 40 demonstrated the efficacy of melatonin in migraine prevention, where it was compared with amitriptyline and a placebo. Their findings revealed that melatonin not only effectively reduced headache frequency but also had a better tolerability profile than amitriptyline, thus enhancing patient compliance for long-term preventive treatment strategies.

Moreover, melatonin's ability to synchronize the body's internal clock is vital in addressing the comprehensive needs of MAMS management. By improving sleep quality, which is often compromised in individuals with headaches, anxiety, and mood disorders, melatonin tackles a crucial aspect of these conditions. This dual-action capability of improving sleep and preventing migraines positions melatonin as an integral part of integrative treatment strategies for MAMS, underscoring the need for further research and its inclusion in treatment plans for a more holistic approach and improved patient outcomes.

In conclusion, effective treatment of PHs involves an approach that extends beyond pain management. A comprehensive understanding of the interplay between sleep, anxiety, and mood disorders is crucial for therapeutic success. Employing a symptom-based approach can significantly benefit patients suffering from these interconnected conditions. Future research, particularly papers targeting psychiatrists and sleep specialists, is essential. It is imperative to encourage studies that concurrently address these conditions.
